# Patterns of nucleotide diversity and phenotypes of two domestication related genes (*OsC1* and *Wx*) in indigenous rice varieties in Northeast India

**DOI:** 10.1186/1471-2156-15-71

**Published:** 2014-06-16

**Authors:** Baharul Islam Choudhury, Mohammed Latif Khan, Selvadurai Dayanandan

**Affiliations:** 1 Forest and Evolutionary Genomics Laboratory and Centre for Structural and Functional Genomics, Biology Department, Concordia University Montréal, 7141 Sherbrooke St. West, Montréal, Quebec H4B 1R6, Canada; 2 Québec Centre for Biodiversity Sciences, Montréal, QC, Canada; 3 Department of Botany, Dr. Hari Singh Gour Central University, Sagar 470003, Madhya Pradesh, India

**Keywords:** Indigenous, Nucleotide diversity, NE India, Rice, Trait specific genes

## Abstract

**Background:**

During the domestication of crops, individual plants with traits desirable for human needs have been selected from their wild progenitors. Consequently, genetic and nucleotide diversity of genes associated with these selected traits in crop plants are expected to be lower than their wild progenitors. In the present study, we surveyed the pattern of nucleotide diversity of two selected trait specific genes, *Wx* and *OsC1*, which regulate amylose content and apiculus coloration respectively in cultivated rice varieties. The analyzed samples were collected from a wide geographic area in Northeast (NE) India, and included contrasting phenotypes considered to be associated with selected genes, namely glutinous and nonglutinous grains and colored and colorless apiculus.

**Results:**

No statistically significant selection signatures were detected in both *Wx* and *OsC1*gene sequences. However, low level of selection that varied across the length of each gene was evident. The glutinous type varieties showed higher levels of nucleotide diversity at the *Wx* locus (π_tot_ = 0.0053) than nonglutinous type varieties (π_tot_ = 0.0043). The *OsC1* gene revealed low levels of selection among the colorless apiculus varieties with lower nucleotide diversity (π_tot_ = 0.0010) than in the colored apiculus varieties (π_tot_ = 0.0023).

**Conclusions:**

The results revealed that functional mutations at *Wx* and *OsC1*genes considered to be associated with specific phenotypes do not necessarily correspond to the phenotypes in indigenous rice varieties in NE India. This suggests that other than previously reported genomic regions may also be involved in determination of these phenotypes.

## Background

The domestication of plants and animals is considered as one of the most important events in the human history that increased the food security to support increasing human population. The process of domestication involves selection of individuals from wild progenitors to fulfill human needs [1]. The Asian cultivated rice is one of the earliest domesticated crop species selected for many traits relevant for human consumption and large-scale agriculture. The most important domestication related traits and corresponding genes identified so far in rice with significant morphological and physiological modifications include reduction in grain shattering [2,3], changes in grain coloration [4], grain size and shape [5], grain fragrance and flavor [6], grain number [7], grain weight [8] and grain stickiness [5]. The genes that control these traits are often called ‘domestication genes’ in crop plants. In addition to human mediated selection for specific traits, the environment where crops grown also may have played a major role in selection and changes in genetic diversity of crop plants.

Domestication is often associated with reduction in genetic variation in domesticated plants as compared to their wild progenitors [1]. This is mainly due to population bottlenecks and artificial selection of domestication genes for desirable traits. Domesticated plants are a product of relatively small founder populations, in which only a sub-sample of the wild progenitor population contributes to the genomes of cultivated plants [9]. As a result, genome-wide loss of genetic variation is found in cultivated plants [1]. The artificial selection targeted to specific desirable traits controlled by domestication genes also reduces the genetic diversity in crop plants as compared to their wild ancestors [10]. Many traits generally suitable for human needs have been targets of selection during the domestication of crops. These traits and associated genes have subsequently undergone changes in response to selection due to local environment and cultural preferences (e.g., grain color, taste) [11]. Thus, analyses of nucleotide sequences of domestication genes at the DNA level are invaluable to gain insights into types of selection that has occurred during domestication.

Several studies have demonstrated the selective sweep in domestication genes and genomic regions in domesticated crops [12-14]. Olsen *et al*. [15] showed one to two fold increase in selection pressure in domestication genes as compared to genes under natural selection. However, the reduction in genetic diversity within various regions of selected genes may vary depending on the relevance of a given region for determining the trait.

Indigenous rice varieties cultivated in the Eastern Himalayan region of NE India are phenotypically diverse and many of which are intricately associated with local cultural and traditional practices. One of the most important culinary and cultural practices found throughout NE India is the use of glutinous rice as a food of choice during festival seasons [16]. Thus, along with nonglutinous rice varieties, numerous glutinous rice varieties are widely cultivated in NE India. The glutinous and nonglutinous nature of rice is primarily determined by the composition of starch in the endosperm tissue. Starch in rice endosperm contains two types of polysaccharides namely amylose and amylopectin. Rice varieties with high amylose levels (~20-30%) tend to form discrete, noncohesive (non-sticky) grains when cooked, whereas varieties with lower amylose levels form cohesive (sticky) cooked grains, commonly known as glutinous [15]. Previous studies have shown that a mutation in the *Waxy* (*Wx*) gene that encodes granule-bound starch synthase drastically reduces (<1%) synthesis of amylose in the endosperm of glutinous rice [17]. The point mutation from G to T at the 5′ splice site of the *Wx* intron 1 is known to cause incomplete post-transcriptional processing of the pre-mRNA in glutinous rice varieties [17-19]. On the other hand, nonglutinous rice varieties possess multiple *Wx* alleles and shows wide variation in amylose content [20]. A highly variable microsatellite (CT_n_) in the 5′ untranslated exon 1 of the *Wx* gene is known to contain many alleles and the size of the allele is correlated with the amylose content in rice varieties [20,21]. Some nonglutinous and low-amylose containing varieties also known to carry the G to T mutation at the 5′ splice site of *Wx* gene suggesting that mutation in the *Wx* gene may not necessarily be responsible for the glutinous phenotype [22-24].

Another morphological variation found among indigenous rice varieties in NE India is the apiculus coloration. The apiculus of the wild ancestor of cultivated rice, *O. rufipogon,* is pigmented whereas apiculus of cultivated rice varieties could be colored or colorless. The colored apiculus phenotype is attributable to anthocyanin pigments, which are known to be associated with coloration in various plant parts. Anthocyanins perform multiple biological functions in plants including protection against UV radiation, defense responses and signal molecules in plant-microbe interactions [25,26]. Saitoh *et al*. [27] identified and mapped the *OsC1* gene in rice responsible for anthocyanin pigmentation and apiculus coloration in rice. Comparative sequence analysis revealed that colorless lines differed from their colored counterpart by a 10-bp deletion located in the R3 repeat located within the third exon of the *OsC1* gene [27].

In this study, we analyzed (a) mutations in *Wx* and *OsC1* genes in indigenous rice varieties in NE India, and their corresponding phenotypes, and (b) nucleotide diversity patterns in these genes across rice varieties to detect selection signatures in domestication related genes. In contrary to expectations, we found greater levels of diversity at the *Wx* gene in glutinous varieties as compared to non-glutinous varieties, and low levels of selection in colourless apiculus varieties, suggesting the existence of other, as-yet unknown genes contributing to these phenotypes.

## Methods

### Plant samples

**Figure 1:**
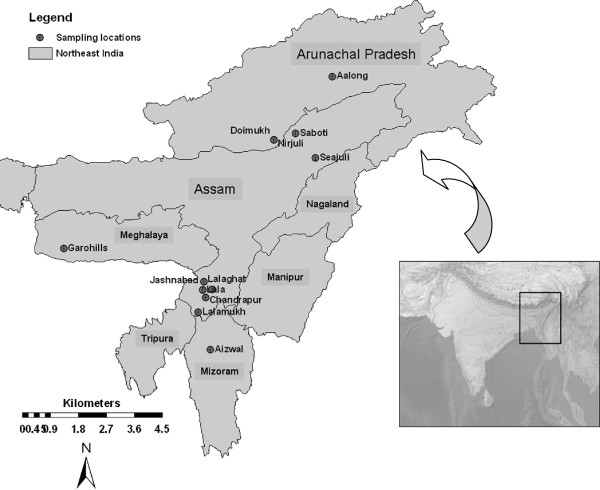
Map showing traditionally cultivated indigenous rice sampling sites in Northeast India.

In the present study, altogether 29 cultivated rice varieties (including 5 agronomically improved varieties) and one wild rice species (*O. rufipogon*) from NE India were included (Figure 1). Two trait specific genes corresponding to contrasting phenotypes were chosen to study. The samples studied included five glutinous and 24 nonglutinous varieties, and 8 colored apiculus and 21 colorless apiculus varieties (Table 1). The wild rice species (*O. rufipogon*), which is nonglutinous and colored apiculus was used as an outgroup. Plant morphology and grain characteristics were noted based on direct observation, interviewing the farmers in the field or records from the International Rice Research Institute (IRRI), Philippines. Seeds were germinated in Petri dishes, transferred to pots and grown in the greenhouse. Leaf samples from seedlings were harvested, air dried, and genomic DNA was extracted following modified cetyltrimethyl ammonium bromide extraction protocol [28,29].

**Table 1:** **Rice variety names, phenotype, and functional mutations at the *Wx *** **and *OsC1 *** **genes**

**Variety**	**Grain quality**	***Wx*** **5′ splice site**	***Wx*** **CTn**	**Apiculus color**	***OsC1*** **10 bp deletion**
Bas Beroin	Glutinous	T	17	Colored	No
Til Bora	Glutinous	T	17	Colored	No
Ranga Borah	Glutinous	G	11	Colorless	Yes
Kakiberoin	Glutinous	G	11	Colorless	Yes
Borua Beroin	Glutinous	T	17	Colorless	No
Joha	Non Glutinous	G	18	Colored	No
Bherapawa	Non Glutinous	G	17	Colored	No
Lallatoi	Non Glutinous	G	11	Colored	Yes
Kawanglawang	Non Glutinous	T	17	Colored	No
Hati Hali	Non Glutinous	G	18	Colored	No
Balam	Non Glutinous	G	11	Colored	No
Bashful	Non Glutinous	G	10	Colorless	No
Lahi	Non Glutinous	G	17	Colorless	No
Borjahinga	Non Glutinous	G	11	Colorless	No
Moircha	Non Glutinous	G	11	Colorless	Yes
Aubalam	Non Glutinous	G	11	Colorless	Yes
Papue	Non Glutinous	G	20	Colorless	Yes
Sorpuma	Non Glutinous	G	10	Colorless	Yes
Mimutim	Non Glutinous	G	18	Colorless	Yes
Local Basmati	Non Glutinous	G	11	Colorless	Yes
Arfa	Non Glutinous	G	11	Colorless	Yes
Mulahail	Non Glutinous	G	10	Colorless	Yes
Guaroi	Non Glutinous	G	17	Colorless	Yes
Harinarayan	Non Glutinous	G	17	Colorless	Yes
Ranjit	Non Glutinous	G	11	Colorless	Yes
IR8	Non Glutinous	G	11	Colorless	Yes
Bahadur	Non Glutinous	G	11	Colorless	Yes
Pankaj	Non Glutinous	G	12	Colorless	Yes
Joya	Non Glutinous	G	11	Colorless	Yes
*O. rufipogon*	Non Glutinous	G	7	Colored	No

*Abbreviations: Wx* waxy gene, *CT*_*n*_ number of CT repeat.

### Loci studied, PCR amplification and sequencing

**Figure 2:**
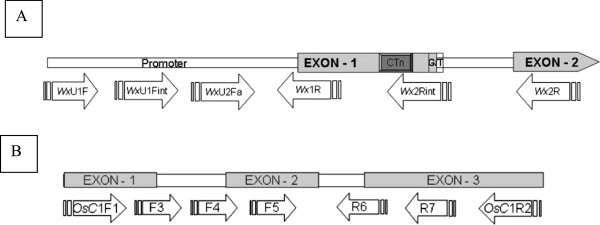
**The locations of the coding and non-coding regions of *Wx *(A) and *OsC1 *(B) genes.** Arrows at the bottom indicate primers used for PCR amplification.

We analyzed nucleotide polymorphism in two trait specific genes, waxy (*Wx*), the gene associated with granule bound starch synthesis and *OsC1*, the gene associated with anthocyanin biosynthesis and apicule coloration. Nucleotide sequences of oligonucleotide primers used for amplification and sequencing are given in Table 2. A portion of the *Wx* gene (~2.7-kb region) surrounding previously identified intron 1 splice donor site mutation, promoter sequence, entire exon 1, intron 1, the 5′ end of exon 2, and the entire noncoding region within exon 2 (Figure 2A) were sequenced following the protocol of Olsen and Purugganan [24]. The *OsC1* gene region (~1.3-kb region) (Figure 2B) was amplified and sequenced following Saitoh *et al*. [27].

**Table 2:** List of genes surveyed and primer sequences used in the study

**Gene name**	**Primer name**	**Primer sequence (5′ - 3′)**	**Functional association**
Waxy [24]	WxU1F	GCCGAGGGACCTAATCTGC	Granule-bound starch synthase
	Wx1R	TGGTGTGGGTGGCTATTTGTAG	
	Wx2FaF	GCCCCGCATGTCATCGTC	
	Wx2R	GTTGTCTAGCTGTTGCTGTGGA	
	Wx1Fint	TTGTCAGCACGTACAAGCA	
	Wx2Rint	GCTATATACATTTTCCTTTGACCAA	
*OsC1*[27]	OsC1F1	ATCGCTCAGTCTCACACCGCA	Anthocyanin biosynthesis
	OsC1F3	GAGGGA GAATGGGGAGGAGAGC	
	OsCF4	TAATTGTGATCTGTATGGATGCTG	
	OsC1F5	GATCGATCGTGTATATATGTTGTCAGGT	
	OsC1R6	GTTGCTGTGTCGGTGT CGGCG	
	OsC1R7	ATGGCCGTCTCCTAATTCCCCTGC	
	OsC1R2	CGTACGGACGACGAACTAATGTCAC	

PCR amplifications were performed in an Applied Biosystems thermal cycler in a total volume of 25 μL reaction mixture consisting of 0.25 mM dNTP, 2.0 mM MgCl_2_, 2.5 μL of 10X buffer, 1.5 pmol of each primer and 0.2 U *Taq* polymerase. The thermal cycling profiles as described in previous publications (*Wx*: [24], and *OsC1*: [27]) were followed. The amplified DNA products were separated through electrophoresis on 1% agarose gels containing with 0.33 μg/ml ethidium bromide. The electrophoresis was performed at 90 V for 40 minutes in a 24 cm long electrophoretic apparatus containing 1 X TBE electrode buffer. DNA fragments on agarose gels were visualized using an ultraviolet (302 nm) transilluminator (UVP Inc), and the size of the amplified DNA fragments was determined using GeneRuler 1 kb DNA ladder (Fermentas) as a size standard. The PCR products were sequenced after purification using Bio-Basic PCR product purification kit (Bio-Basic inc.).

### Data analysis

DNA sequence chromatograms were analyzed using the software program Geneious version 5.4.6 (http://www.geneious.com/) and visually inspected for ambiguities. The resulting consensus DNA sequences were aligned using the software program ClustalW v2 [30]. The coding and non-coding regions of the gene were identified by comparing with annotated DNA sequences of corresponding genes downloaded from the GenBank.

In order to examine the patterns of nucleotide diversity resulting from evolutionary changes in DNA sequences in relation to neutral expectations and signatures of selection due to domestication process, several analyses as described below were performed using the software program DnaSP version 5.1 [31]. The θ_w_ based on the number of segregating sites [32], π based on mean pairwise nucleotide differences among sequences [33], Tajima’s *D*[34], Fu and Li’s D* and F* [35] were calculated, and McDonald and Kreitman [36] analysis was performed. D* and F* are more sensitive than Tajima’s *D* in detecting deviations from neutrality based on low-frequency polymorphisms, population expansion and positive selection [35]. The McDonald and Kreitman [36] test is insensitive to demographic histories and geographic structuring of the populations. Thus, use of a variety of approaches that differ in underlying assumptions provides a means to discern the historical processes associated with shaping the patterns of nucleotide diversity. The changes in nucleotide diversity and associated statistic in different regions of the gene was examined using the sliding-window analysis approach. The rates of synonymous (*dS*) and non-synonymous (*dN*) substitution in each of the selected genes among different rice types were calculated. The ratio of *dN/dS* provides an insight into the long-term selection pressure and purifying selection during the domestication process. Number of haplotypes was calculated and the haplotype network diagram was constructed using NETWORK 4.5.1 (Fluxus Technology Ltd. at http://www.fluxus-engineering.com).

## Results

A total of 53 indel polymorphisms with an average length of 3.525 were detected from the two sequenced regions (Table 3). The size of indels varied in length and ranged from one to 20 nucleotides in both coding and noncoding regions. Single nucleotide polymorphisms (SNP) were more frequent than indels. Total numbers of SNPs found among the sequenced regions were 91 with an average of 1 SNP at every 44.33 nucleotides.

**Table 3:** Lengths of aligned nucleotide sequences (bp) and site categories

**Gene region**	**Total length including indels**	**Total no. of sites excluding indels**	**No. of indels**	**No. of indel polymorphisms**	**Length of coding region excluding indels**	**Length of coding region including indels**	**Length of noncoding region excluding indels**	**Length of noncoding region including indels**	**SNP**
Waxy	2770	2574	195	50	177	197	2574	2593	84
*OsC1*	1296	1284	12	3	809	824	475	476	7

### Polymorphism of the *Wx* gene

The aligned length, including both coding and non-coding regions of the *Wx* gene was 2770 nucleotides. A total of 50 indels were detected with an average length of 2.12 nucleotides across all samples. The exon 1 (5′ untranslated region) of the *Wx* gene contained a highly variable microsatellite (CT_n_). A total of seven alleles of this microsatellite (n = 7, 10, 11, 12, 17, 18, and 20) were detected among rice varieties included in the present study. Alleles CT_10_, CT_11_, CT_17_, and CT_18_ were found in 3, 13, 8 and 3 cultivated varieties respectively. The CT_12_ and CT_20_ alleles were found in one cultivated variety each. A unique CT_7_ allele was found in the wild rice *O. rufipogon*. The number of SNPs was higher than the number of indels, with a total of 84 SNPs resulting in average 1 SNP for 32.98 bp among all samples. Relatively fewer SNP (1) and indels (6) were found in glutinous varieties than in the nonglutinous varieties (17 indels and 7 SNPs). The total number of mutations was also higher among the nonglutinous varieties than in the glutinous varieties (Table 3).

The G to T mutation at the 5′ splice donor site of the *Wx* intron 1, which is known to be associated with drastic reduction in amylose synthesis in glutinous rice varieties [17] was not consistently present among glutinous rice varieties included in the present study. The results revealed that T nucleotide was present in four varieties, while G nucleotide was found in the remaining 25 cultivated rice varieties and in the wild rice. The T nucleotide was found in three of the five glutinous varieties (*Borua Beroin*, *Bas Beroin* and *Til Bora*), and G nucleotide was present in other two glutinous (*Ranga Borah* and *Kakiberoin*) varieties. On the contrary, the T nucleotide at this site was found in one of the nonglutinous (*Kawanglawang*) varieties.

**Figure 3:**
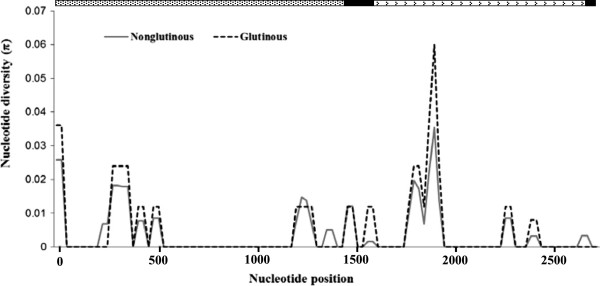
**Nei’s Nucleotide diversity (π) patterns along *Wx *gene in sliding window among glutinous and nonglutinous grain types.** Analysis was performed using a window length of 50 bp and steps of 25 bp. ( promoter region;  exon;  intron).

The nucleotide diversity analyses results showed that nucleotide diversity of glutinous varieties was higher (π_tot_ = 0.0053; θ_tot_ = 0.0043) than the nonglutinous varieties (π_tot_ = 0.0043; θ_tot_ = 0.0033). The sliding window analysis of the *Wx* gene revealed high nucleotide diversity at three regions located at 1 to 600, 1150 to 2000 and 2300 to 2500 bp of the gene. This analysis further revealed that polymorphic sites were mostly located at the beginning and end of the promoter region, the exon 1 carrying the microsatellite and the first part of intron 1 (Figure 3).

### Neutrality analysis at the *Wx* locus

**Figure 4:**
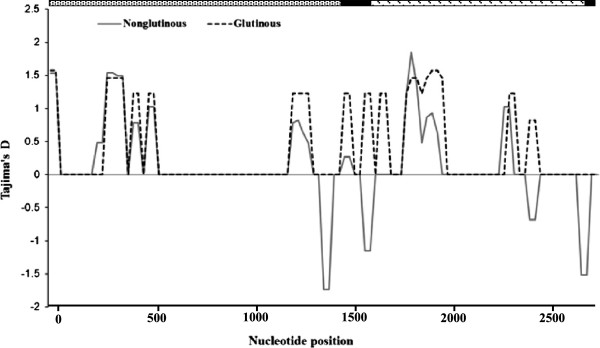
**Tajima’s *D *statistics in sliding window analysis for the *Wx *gene among rice ecotypes and glutinous and nonglutinous rice varieties.** Computation was performed using a window length of 50 bp and steps of 25 bp ( promoter;  exon;  intron).

The values of Tajima’s *D* and Fu and Li’s D* and F* based on the *Wx* locus were not significantly different from neutral expectations. The values of *D* or D* and F* were positive for glutinous and nonglutinous varieties at the *Wx* locus (Table 4), indicating a weak overdominant selection or population size reduction. The sliding window analyses of Tajima’s *D* showed that glutinous varieties had only positive values while nonglutinous varieties had both positive and negative values at different regions of the gene (Figure 4). Negative *D* values were detected in the regions between 1357–1432, 1575–1655, 2400–2476, 2659–2735 bp only in nonglutinous varieties. These regions are located in the intron-1 and 2 and the exon-1 of the *Wx* gene. The observed pattern of variability is not significantly different from expected variability under the neutral model of evolution and neutrality hypothesis cannot be rejected. The McDonald and Kreitman test did not show departure from neutrality for the glutinous and non-glutinous varieties (Table 4) indicating no signature of selection at the *Wx* locus.

**Table 4:** Levels of nucleotide variation at the two studied genes

**Gene**	**Ecotype**	**Indel**	**SNP**	**S**	**π**_**tot**_	**θ**_**tot**_	**dN/dS**	***D***	***D*** *	***F*** *
*Wx*	Glutinous	6	1	23	0.0053	0.0043	-	1.7295	1.7295	1.8583
	Nonglutinous	17	7	31	0.0043	0.0033		1.1825	0.9145	1.369
*OsC1*	Colored	2	1	6	0.0023	0.0020	-	0.8109	1.0088	1.1449
	Colorless	3	8	10	0.0010	0.0021	1.00	−1.7683	−1.2847	−1.7178

S, number of segregating sites; π, average number of nucleotide differences per site between two sequences [33] calculated on the total number of polymorphic sites (π_tot_); silent sites (π_sil_); synonymous sites (π_syn_); nonsynonymous sites (π_nonsyn_); θ, Watterson’s estimator of nucleotide polymorphism per base pair [32] calculated on the total number of polymorphic sites (θ_tot_); silent sites (θ_sil_); synonymous sites (θ_syn_); nonsynonymous sites (θ_nonsyn_); D, Tajima’s *D*[34]; D*, Fu and Li’s D*; F*, Fu and Li’s F* [35].

Tajima’s *D*, *Fu and Li’s D* and F* not significant (P > 0.10).

**Figure 5:**
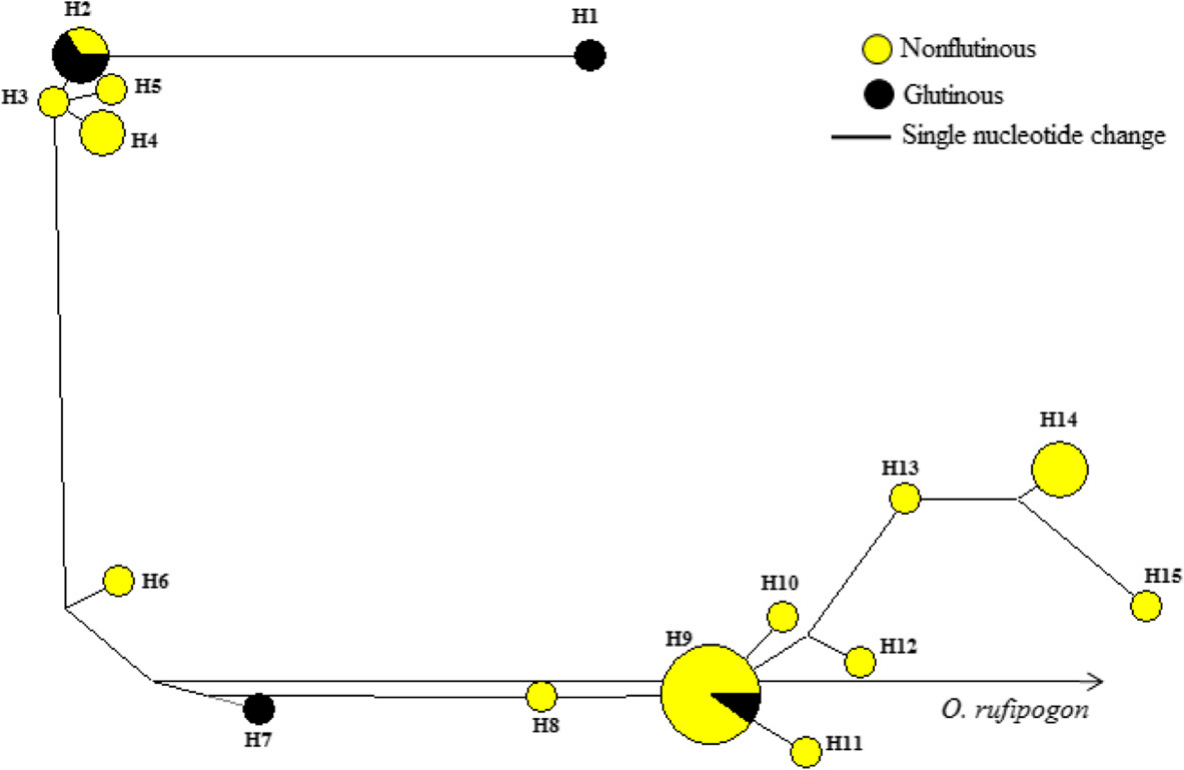
**Haplotype network based on *Wx *** **gene.**

The analyses of SNPs revealed 16 distinct *Wx* haplotypes among studied rice varieties including the wild rice (Figure 5) and formed two distinct groups (haplotypes 1–5 and haplotypes 6–15). One variety each consisting of two haplotypes (H1 and H7) were glutinous type. Two varieties with the haplotype H2 and one variety with the haplotype H9 were glutinous type. The analyses based on SNPs and indels together revealed 28 haplotypes, and indel only analyses revealed 26 haplotypes among the studied samples.

### Polymorphism at the *OsC1* gene

The aligned *OsC1* gene region was 1296 bp long and included both exons and introns. The results of the present study showed that 62% of the sequenced samples contained the 10 bp deletion in the R3 repeat region of the *OsC1* gene known to cause a frameshift leading to colorless apiculus in rice [27]. In agreement with the expected phenotype of the genotype, the 10 bp deletion was found in 17 colorless apiculus varieties included in the present study and the corresponding deletion was absent in seven colored apiculus varieties and *O. rufipogon* (Table 1). However, there were incongruences between the genotype and the phenotype of several varieties examined in the present study. The 10 bp deletion was not found in four colorless apiculus varieties (*Bashful*, *Borua Beroin*, *Lahi* and *Borjahinga*), and the corresponding 10 bp deletion was found in one of the colored apiculus varieties (*Lallatoi*).

Three non-synonymous substitutions were detected in the coding regions of the *OsC1* gene. One single nucleotide polymorphism (SNP) was detected in the exon-1 with a mutation of G to C at the position 60 resulting in an amino acid change from positively charged Lysine to negatively charged Aspartic acid. Another SNP was detected in the exon-1 with a mutation of C to G at the position 122 in the variety *Bashful*, resulting in an amino acid change of non-polar Proline to positively charged Arginine. The other non-synonymous substitution was at the position 845 in the exon 3 with a mutation of G to T resulting in an amino acid change of Alanine to Valine (both hydrophobic). Other than these, eight SNPs were detected in the intronic regions of the *OsC1* gene among different cultivated varieties and wild rice.

**Figure 6:**
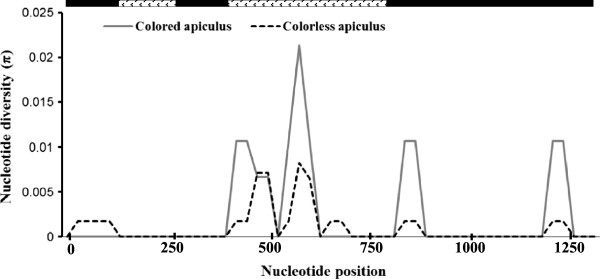
**Nei’s Nucleotide diversity (π) patterns along *OsC1 *gene in sliding window among colored and colorless apiculus rice grains apiculus in rice.** Analysis was performed using a window length of 50 bp and steps of 25 bp. (■ exon;  intron).

The analyses of nucleotide sequences of the *OsC1* gene revealed three indels (average 3.22 bp long) and seven SNPs (average one SNP for every 185.14 bp) among sequenced samples. More indels and SNPs were found in colorless apiculus varieties than in the colored apiculus varieties (Table 4). However, the nucleotide diversity (π: [33]) was higher in the colored apiculus rice varieties than in the colorless apiculus varieties (Table 4). The sliding window analysis of the *OsC1* gene showed that parts of the intron 2 and exon 3 at 400 to 625, 800 to 900 and 1050 to 1250 bp are polymorphic, and the nucleotide diversity in colored apiculus varieties are higher than the colorless apiculus rice varieties (Figure 6).

### Neutrality analysis

**Figure 7:**
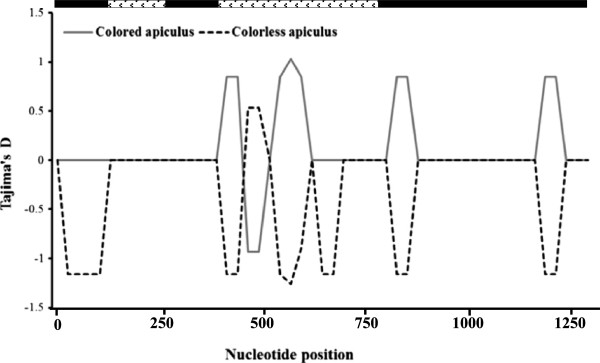
**Tajima’s D statistics in sliding window analysis for the *OsC1 *gene among the colored and colorless apiculus rice grains.** Computation was performed using a window length of 50 bp and steps of 25 bp. (■ exon;  intron).

**Figure 8:**
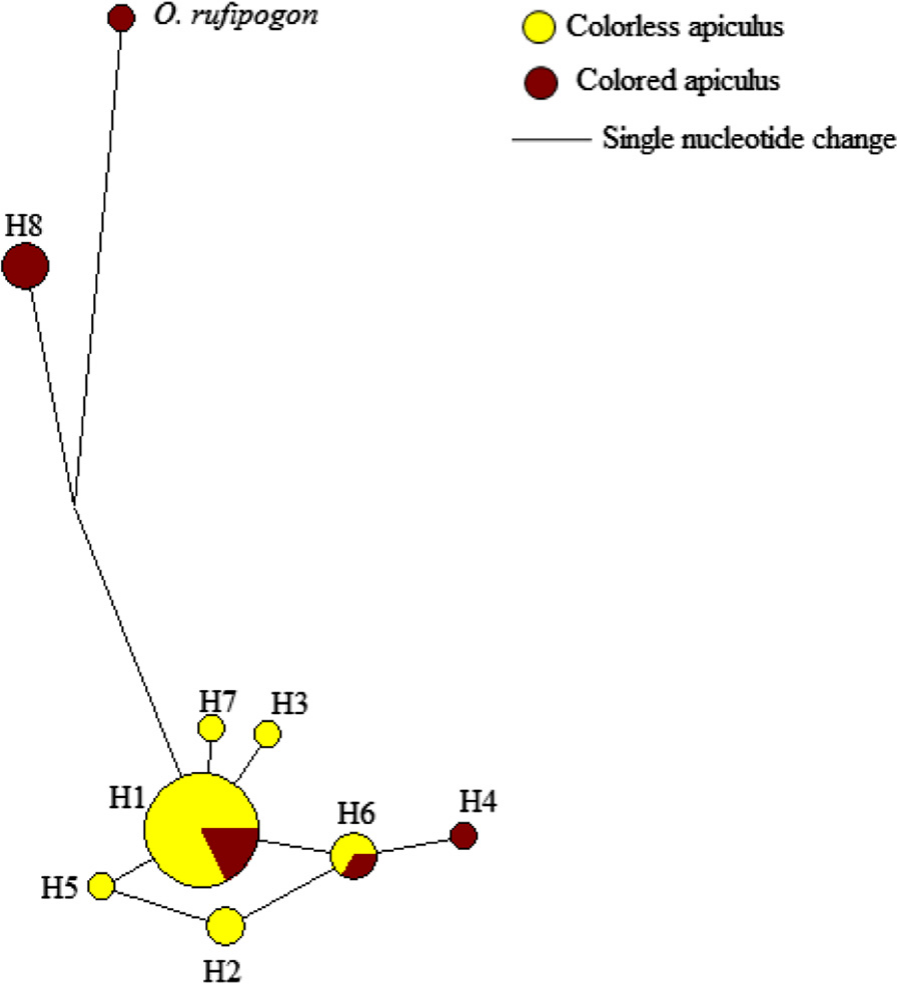
**Haplotype network based on *OsC1 *** **gene.**

The overall values of Tajima’s *D* and Fu and Li’s D* and F* were negative in colorless apiculus rice varieties, and positive in colored apiculus varieties (Table 4). The sliding window analyses of Tajima’s *D* showed mostly negative values in colorless apiculus varieties and mostly positive values in the colored apiculus rice varieties (Figure 7). These values were not significantly different from neutral expectations. The negative D values in colorless apiculus varieties were detected at 25–150, 400–475, 525–700, 811–886 and 1161–1237 bp positions, and a positive value was observed at 475–525 bp position. On the contrary, colored apiculus varieties showed positive *D* values in most regions (400–475, 525–625, 811–886 and 1161–1237 bp) and negative values at the 475–525 bp region (Figure 7). In general, the colorless apiculus varieties showed negative D values in the exon-1, intron-2 and exon-3, and positive D value in the intron-2. Interestingly, an opposite trend was observed in colored apiculus varieties with positive *D* values in intron-2 and exon-2 and negative *D* in value in intron-2.These *D* values, which are not significantly different from neutral expectations indicates that neutrality hypothesis in the *OsC1* gene region cannot be rejected. The McDonald and Kreitman test did not show evidence of selection in the *OsC1* gene (Table 5). Altogether nine haplotypes were detected in the *OsC1* gene (Figure 8). Haplotypes H8 (three varieties) and H4 (one variety) were found only in colored apiculus varieties while haplotypes H1 and H6 were found in both colored and colorless apiculus varieties. Other haplotypes were found only in colorless apiculus varieties. The analyses based on SNPs and indels yielded 15 haplotypes and analyses of indel polymorphisms yielded seven haplotypes among the colored and colorless apiculus rice varieties.

**Table 5:** **McDonald-Kreitman test for the *Wx *** **and *OsC1 *** **genes between different types and *O. rufipogon***

**Locus**	**Ecotypes and grain qualities**	**Silent**		**Non synonymous**	
		^**a**^**Fixed**	**Polymorphic**	**Fixed**	**Polymorphic**
*Wx*	Glutinous	80	22	2	2
	Nonglutinous	80	25	2	3
*OsC1*	Red apiculus	3	6	1	0
	Colorless apiculus	3	8	1	2

^a^Fixed differences in comparison with *O. rufipogon*.

## Discussion

The present study reports the findings of the analyses of DNA sequence variability of two trait specific genes in indigenous rice varieties in the Eastern Himalayan region of NE India. The *Wx* gene is associated with amylose synthesis, which determines the glutinous or nonglutinous nature of rice grains. The *OsC1* gene is involved in the synthesis of anthocyanin and associated with coloration of the apiculus in rice grains. Rice varieties used in this study include glutinous and nonglutinous as well as colored and colorless apiculus types collected from a broad geographic area covering most of the NE India.

The present study revealed that previously identified mutations do not exclusively contribute to the corresponding phenotypes in rice varieties. For example, the glutinous nature in most rice varieties is considered to be a result of a G to T mutation at the 5′ splice donor site of exon 2 of the *Wx* gene [18,22]. In the present study, three of the five glutinous rice varieties carried the G to T mutation at the *Wx* gene, while this mutation was not detected in two of the five glutinous rice varieties. On the other hand, one of the 25 non-glutinous rice varieties carried the G to T mutation, while maintaining the non-glutinous phenotypes. This finding suggests that alternative genes or genomic regions other than the ones previously reported are associated with the glutinous and nonglutinous phenotype of the cultivated rice. Similarly, several reports indicated a correlation between variation in amylose content and the number of repeats in the microsatellite region within the *Wx* gene [37,38]. Although the present study also reports the occurrence of highly variable microsatellite locus within the *Wx* gene, there was no direct correlation between the number of repeats and the glutinous nature of rice grains.

Analyses of the *OsC1* locus also revealed similar patterns. The colorless apiculus in rice varieties is often attributed to a 10 bp deletion in the *OsC1* gene [27]. Although 17 of 21 varieties with colorless apiculus included in the present study had the 10 bp deletion in the *OsC1* gene, five varieties without the corresponding 10 bp deletion showed the colorless phenotype. Similarly, eight varieties without the 10 bp deletion showed colored apiculus phenotype as expected, whereas one of the varieties with the 10 bp deletion showed the colored apiculus phenotype. Thus, apiculus color phenotype of 18% of indigenous rice varieties in NE India did not correspond to the reported apiculus color determining genotype of the *OsC1* gene.

One of the varieties with colorless apiculus phenotype (*Mimutim*) had the 10 bp deletion in the R3 region, and showed the G to C nucleotide change resulting a substitution from Lysine to Aspartic acid possibly contributing to the observed colorless phenotype. Another colorless apiculus variety (*Bashful*) without the 10 bp deletion showed an amino acid change from Proline to Arginine in exon-1 suggesting that this mutation could be associated with the coloration of the apiculus. However, the other three colorless apiculus varieties (*Borua Beroin*, *Lahi* and *Borjahinga*), which lack the 10 bp deletion in exon-3, did not carry the Proline to Arginine amino acid change suggesting that other genomic regions also play a role in determination of the phenotype of the apiculus color. The mutation at the position 845 of the exon-3, which substitutes Alanine to Valine in three varieties and (*Tilbora*, *Kawanglawang* and *Balam*) and *O. rufipogon* showed no effect on the phenotype of the apiculus color, suggesting that the substitution of an amino acid with similar hydrophobicity at this position does not affect the apiculus color phenotype. Overall, these observations suggest that multiple genomic regions are involved in determining a particular phenotype. There are several examples of involvement of multiple genes or interacting loci in determination of the phenotype [24,39,40]. Two of the SNPs, C to G mutation at position 122 in exon 1 and G to T mutation at position 845, have already been identified in a previous study [27]. The G to C mutation at position 60 in exon 1 is reported for the first time in this study.

It is generally considered that the domestication process reduces the nucleotide diversity at domestication related genes that control specific traits selected during the domestication. In other words, genes that regulate a particular trait under positive selection during domestication and improvement process may imprint ‘signatures of selection’ in the form of typical patterns of reduced nucleotide diversity [10]. This is evidenced by much lower levels of nucleotide diversity among glutinous rice at the *Wx* gene as compared to the nonglutinous rice varieties [24,41]. Similar observations of reduced levels of nucleotide sequence polymorphism in the nonshattering *sh4* allele in the cultivated rice varieties as compared to wild progenitors [42], and reduced diversity in the *ramosa1* gene in cultivated maize as compared to the wild teosintes that control branching architecture in the tassel and ear [43] have been reported. However, the present study revealed higher levels of nucleotide diversity (π_tot_ = 0.0053) in the glutinous type varieties than in the nonglutinous type varieties (π_tot_ = 0.0043) at the *Wx* locus. This could be attributable to the fact that *Wx* gene, which has been associated with the glutinous nature of rice, may not be the sole gene that determines the glutinous phenotype. This phenotype is likely controlled by multiple loci. This finding is is further supported by the fact that the *Wx* intron 1 splice donor site mutation (G to T) is also found in some nonglutinous rice varieties reflecting that this mutation is not necessarily responsible for the expression of glutinous phenotype [5,44]. These findings are in agreement with other studies, which showed that interaction of other genes (e.g. dull genes) may modify the phenotype of the *Wx* gene [45] or other dull genes [46]. Teng et al. [47] suggested that allelic variation at *Wx* gene may not necessarily regulate the starch properties in different rice varieties. The linkage association study also showed an interplay of multiple genes in determining starch physicochemical properties in rice [48].

Although selective sweeps may drastically reduce nucleotide diversity in target genes such as *Wx* locus [15], the diversifying selection due to environmental heterogeneity and local cultural preferences favoring other traits may increase nucleotide diversity [49]. The existence of diverse agroclimatic conditions, and various cultural practices of indigenous communities may have played a significant role in the maintenance of high levels of diversity in glutinous varieties of rice in NE India.

In the present study, positive values of Tajima D values were detected for the glutinous and non-glutinous varieties (Table 4) except for small regions of the *Wx* gene that showed negative values among nonglutinous varieties (Figure 4). Since the values of Tajima’s *D* were not significantly different from zero, the overall distribution of nucleotide diversity falls within the neutral expectations (Table 4). Since demographic changes including population expansion or reduction may influence all regions of the genome equally, the differences in Tajima D within and between loci could be attributable to selection trends during the domestication process. Therefore, regions of the gene that shows positive Tajima D value could be attributable to balancing or overdominant selection, whereas the regions of gene with negative Tajima D value could be associated with the purifying selection. Signature of positive selection shown in McDonald and Kreitman test at the *Wx* gene may be linked to some traits of ecological adaptation into diverse agroclimatic conditions. The deviations detected in various analyses are not significantly different from neutral expectations and conforms that selection pressure associated with both traits are weak. Similar results have also been reported in previous studies in rice [24] and maize [13,14]. The total of 16 haplotypes detected at the *Wx* locus is lower than the previously reported 18 haplotypes among 37 glutinous and 68 nonglutinous rice accessions from Asia [24]. However, the 16 haplotypes reported in our study are different than haplotypes found in the previous study. There was no clear haplotype based partitioning of the rice varieties into glutinous and nonglutinous varieties. Haplotype analysis based on *Wx* locus showed that haplotypes H1 to H5 formed a distinct cluster consisting of only indigenous varieties and could serve as a valuable material for future genetic improvement programs. Although number of haplotypes varied when indels were considered in the network analysis, there was no clear grouping based on phenotypes.

The *OsC1* gene showed lower levels of polymorphism and reduced nucleotide diversity among the colorless apiculus varieties as compared to colored apiculus varieties. The low level of nucleotide diversity is common in genes related to selected phenotypes [24,42]. Sliding window analysis of the nucleotide diversity showed that most regions of reduced nucleotide diversity in *OsC1* gene were same between colored and colorless apiculus phenotypes (Figure 6). Such concordant loss of diversity could be attributable to population bottleneck during the domestication [50].

The evidence for selection among colorless apiculus varieties is detected through high dN/dS ratio at the *OsC1* locus (Table 4). As this gene is associated with synthesis of anthocyanins, which has multiple functions including plant defense responses and signalling in plant-microbe interactions [25,26], selection of this gene among the cultivated rice varieties can not be ruled out. The negative values of the Tajima *D* values indicate an excess of rare alleles (Table 4) at the *OsC1* locus among the colorless apiculus varieties suggesting a possibility of purifying selection. It has been found that colorless apiculus varieties possessed more negative *D* values in the coding regions compared to the colored apiculus counterpart. These patterns are consistent with a recent selective sweep at the *OsC1* gene among the colorless apiculus rice varieties. Translation of the coding regions of *OsC1* gene revealed that the sequences with the 10-bp deletion within the third exon drastically reduces the protein size from 272 amino acid to 206 amino acid. This might have significant impact in expression of the *OsC1* gene and regulation of apiculus coloration in rice.

The haplotype analysis revealed nine different haplotypes among the colored and colorless apiculus varieties. The number of detected haplotypes is about 50% less than the previously reported haplotypes (17) among 39 wild and cultivated rice [27]. On the other hand, only two haplotypes reported in Saitoh et al. [27] were detected in our samples and the remaining seven haplotypes were unique to our study. These haplotypes formed two major groups of rice varieties. However this grouping did not correspond to apiculus coloration. Similar results were also obtained when gaps were included in in the analysis. One group showed affinity with the agronomically improved varieties and the other group consisting of only indigenous varieties formed a separate cluster.

## Conclusion

The present study based on two trait specific genes, *Wx* and *OsC1* reported to be associated with amylose content and apiculus coloration respectively, showed that mutations considered to be associated with a given phenotype of the trait do not necessarily correspond to those phenotypes in indigenous rice varieties in NE India. This suggests that alternative genomic regions also involved in controlling the amylose content and apiculus coloration in rice. Although statistically significant signatures of selection were not detected in both genes, low level of selection that varied across the length of each gene was evident.

Availability of supporting data: Nucleotide sequences reported in this paper has been submitted with the GenBank with accession numbers KJ934819 - KJ934878. The sequences have also been submitted to LabArchives and can be accessed from the following link (DOI 10.6070/H4H41PDH).

## Competing interests

The authors declare that they have no competing interests.

## Authors’ contributions

BIC, MLK and SD contributed to the conceptual development of the study. BIC carried out the molecular genetic work and data analyses guided by SD. BIC, MLK and SD drafted the manuscript. All authors read and approved the final manuscript.

## References

[B1] Doebley JF Gaut BS Smith BD The molecular genetics of crop domestication Cell 2006 127 7 1309 1321 17190597 10.1016/j.cell.2006.12.006

[B2] Konishi S Izawa T Lin SY Ebana K Fukuta Y Sasaki T Yano M An SNP caused loss of seed shattering during rice domestication Science 2006 312 5778 1392 1396 16614172 10.1126/science.1126410

[B3] Li C Zhou A Sang T Rice domestication by reducing shattering Science 2006 311 5769 1936 1939 16527928 10.1126/science.1123604

[B4] Sweeney MT Thomson MJ Pfeil BE McCouch S Caught red-handed: Rc encodes a basic helix-loop-helix protein conditioning red pericarp in rice Plant Cell 2006 18 2 283 294 16399804 10.1105/tpc.105.038430 PMC1356539

[B5] Yamanaka S Nakamura I Watanabe KN Sato YI Identification of SNPs in the waxy gene among glutinous rice cultivars and their evolutionary significance during the domestication process of rice Theor Appl Genet 2004 108 7 1200 1204 14740088 10.1007/s00122-003-1564-x

[B6] Bradbury LM Fitzgerald TL Henry RJ Jin Q Waters DL The gene for fragrance in rice Plant Biotechnol J 2005 3 3 363 370 17129318 10.1111/j.1467-7652.2005.00131.x

[B7] Ashikari M Sakakibara H Lin S Yamamoto T Takashi T Nishimura A Angeles ER Qian Q Kitano H Matsuoka M Cytokinin oxidase regulates rice grain production Science 2005 309 5735 741 745 15976269 10.1126/science.1113373

[B8] Song XJ Huang W Shi M Zhu MZ Lin HX A QTL for rice grain width and weight encodes a previously unknown RING-type E3 ubiquitin ligase Nat Genet 2007 39 5 623 630 17417637 10.1038/ng2014

[B9] Eyre-Walker A Gaut RL Hilton H Feldman DL Gaut BS Investigation of the bottleneck leading to the domestication of maize Proc Natl Acad Sci 1998 95 8 4441 4446 9539756 10.1073/pnas.95.8.4441 PMC22508

[B10] Tanksley SD McCouch SR Seed banks and molecular maps: unlocking genetic potential from the wild Science 1997 277 5329 1063 1066 9262467 10.1126/science.277.5329.1063

[B11] Simmonds NW Evolution of Crop Plants 1976 New York: Longman

[B12] Buckler ES Thornsberry JM Kresovich S Molecular diversity, structure and domestication of grasses Genet Res 2001 77 3 213 218 11486504 10.1017/s0016672301005158

[B13] Wang RL Stec A Hey J Lukens L Doebley J The limits of selection during maize domestication Nature 1999 398 6724 236 239 10094045 10.1038/18435

[B14] Tian F Stevens NM Buckler ES Tracking footprints of maize domestication and evidence for a massive selective sweep on chromosome 10 Proc Natl Acad Sci 2009 106 9979 9986 19528660 10.1073/pnas.0901122106 PMC2702805

[B15] Olsen KM Caicedo AL Polato N McClung A McCouch S Purugganan MD Selection under domestication: evidence for a sweep in the rice waxy genomic region Genetics 2006 173 2 975 983 16547098 10.1534/genetics.106.056473 PMC1526538

[B16] Roder W Keoboulapha B Vannalath K Phouaravanh B Glutinous rice and its importance for hill farmers in Laos Econ Bot 1996 50 4 401 408

[B17] Sano Y Differential regulation of waxy gene expression in rice endosperm Theor Appl Genet 1984 68 5 467 473 24257739 10.1007/BF00254822

[B18] Hirano HY Eiguchi M Sano Y A single base change altered the regulation of the Waxy gene at the posttranscriptional level during the domestication of rice Mol Biol Evol 1998 15 8 978 987 9718725 10.1093/oxfordjournals.molbev.a026013

[B19] Isshiki M Morino K Nakajima M Okagaki RJ Wessler SR Izawa T Shimamoto K A naturally occurring functional allele of the rice waxy locus has a GT to TT mutation at the 5′ splice site of the first intron Plant J 1998 15 1 133 138 9744101 10.1046/j.1365-313x.1998.00189.x

[B20] Ayres NM McClung AM Larkin PD Bligh HFJ Jones CA Park WD Microsatellites and a single-nucleotide polymorphism differentiate apparentamylose classes in an extended pedigree of US rice germplasm Theor Appl Genet 1997 94 6–7 773 781

[B21] Wan YX Deng QM Wang SQ Liu MW Zhou HQ Li P Genetic polymorphism of *Wx* gene and its correlation with main grain quality characteristics in rice Rice Sci 2007 14 2 85 93

[B22] Wang ZF Zheng FQ Shen GZ Gao JP Snusted DP Li MG Zhang JL Hong MM The amylose content in rice endosperm is related to the post-transcriptional regulation of the waxy gene Plant J 1995 7 613 622 7742858 10.1046/j.1365-313x.1995.7040613.x

[B23] Cai XL Wang ZY Xing YY Zhang JL Hong MM Aberrant splicing of intron 1 leads to the heterogeneous 5′ UTR and decreased expression of waxy gene in rice cultivars of intermediate amylose content Plant J 1998 14 4 459 465 9670561 10.1046/j.1365-313x.1998.00126.x

[B24] Olsen KM Purugganan MD Molecular evidence on the origin and evolution of glutinous rice Genetics 2002 162 2 941 950 12399401 10.1093/genetics/162.2.941 PMC1462305

[B25] Dooner HK Robbins TP Jorgensen RA Genetic and developmental control of anthocyanin biosynthesis Annu Rev Genet 1991 25 1 173 199 1839877 10.1146/annurev.ge.25.120191.001133

[B26] Koes RE Quattrocchio F Mol JN The flavonoid biosynthetic pathway in plants: function and evolution BioEssays 1994 16 2 123 132

[B27] Saitoh K Onishi K Mikami I Thidar K Sano Y Allelic diversification at the *C* (*OsC1*) Locus of wild and cultivated rice nucleotide changes associated with phenotypes Genetics 2004 168 2 997 1007 15514070 10.1534/genetics.103.018390 PMC1448844

[B28] Doyle JJ Doyle JL A rapid DNA isolation procedure for small quantities of fresh leaf tissue Phytochem Bull 1987 19 11 15

[B29] Dayanandan S Bawa KS Kesseli RV Conservation of microsatellites among tropical trees (Leguminosae) Am J Bot 1997 84 1658 1663 21708569

[B30] Larkin MA Blackshields G Brown NP Chenna R McGettigan PA McWilliam H Valentin F Wallace IM Wilm A Lopez R Thompson JD Gibson TJ Higgins DG Clustal W and Clustal X version 2.0 Bioinformatics 2007 23 21 2947 2948 17846036 10.1093/bioinformatics/btm404

[B31] Librado P Rozas J DnaSP v5: a software for comprehensive analysis of DNA polymorphism data Bioinformatics 2009 25 11 1451 1452 19346325 10.1093/bioinformatics/btp187

[B32] Watterson GA On the number of segregating sites in genetical models without recombination Theor Popul Biol 1975 7 2 256 276 1145509 10.1016/0040-5809(75)90020-9

[B33] Nei M Molecular Evolutionary Genetics 1987 New York: Columbia University Press

[B34] Tajima F Statistical method for testing the neutral mutation hypothesis by DNA polymorphism Genetics 1989 123 3 585 595 2513255 10.1093/genetics/123.3.585 PMC1203831

[B35] Fu YX Li WH Statistical tests of neutrality of mutations Genetics 1993 133 3 693 709 8454210 10.1093/genetics/133.3.693 PMC1205353

[B36] McDonald JH Kreitman M Adaptive protein evolution at the Adh locus in Drosophila Nature 1991 351 6328 652 654 1904993 10.1038/351652a0

[B37] Shu QY Wu DX Xia YW Gao MW Ayres NM Larkin PD Park WD Microsatellites polymorphism on the waxy gene locus and their relationship to amylose content in indica and japonica rice, *Oryza sativa* L Acta Genet Sin 1999 26 4 350 358

[B38] Bao JS Corke H Sun M Microsatellites, single nucleotide polymorphisms and sequence tagged site in starchsynthesizing genes in relation to starch physiochemical properties in non-waxy rice (*Oryza sativa* L.) Theor Appl Genet 2006 113 1185 1196 16964521 10.1007/s00122-006-0394-z

[B39] Doebley J Stec A Wendel J Edwards M Genetic and morphological analysis of a maize-teosinte F2 population: implications for the origin of maize Proc Natl Acad Sci 1990 87 9888 9892 11607138 10.1073/pnas.87.24.9888 PMC55279

[B40] Zhu Y Ellstrand NC Lu BR Sequence polymorphisms in wild, weedy, and cultivated rice suggest seed-shattering locus sh4 played a minor role in Asian rice domestication Ecol Evol 2012 2 9 2106 2113 23139871 10.1002/ece3.318 PMC3488663

[B41] Wei-Hua Q You-Tao C Rong-Sheng W Xin W Li-Rong C Wan-Xia Z Qing-Wen Y Nucleotide diversity in gene and validation of single nucleotide polymorphism in relation to amylose content in Chinese microcore rice germplasm Crop Sci 2012 52 4 1689 1697

[B42] Zhang D Zhang H Wang M Sun J Qi Y Wang F Li Z Genetic structure and differentiation of *Oryza sativa* L. in China revealed by microsatellites Theor Appl Genet 2009 119 6 1105 1117 19649611 10.1007/s00122-009-1112-4

[B43] Sigmon B Vollbrecht E Evidence of selection at the ramosa1 locus during maize domestication Mol Ecol 2010 19 7 1296 1311 20196812 10.1111/j.1365-294X.2010.04562.x

[B44] Inukai T Sako A Hirano HY Sano Y Analysis of intragenic recombination at wx in rice: correlation between the molecular and genetic maps within the locus Genome 2000 43 4 589 596 10984169 10.1139/g00-015

[B45] Zeng M Morris CF Batey IL Wrigley CW Sources of variation for starch gelatinization, pasting, and gelation properties in wheat Cereal Chem 1997 74 63 71

[B46] Kiswara G Lee JH Hur YJ Cho JH Lee JY Kim SY Kim KM Genetic analysis and molecular mapping of low amylose gene du12 (t) in rice (Oryza sativa L.) Theor Appl Genet 2014 127 1 51 57 24114051 10.1007/s00122-013-2200-z

[B47] Teng B Zhang Y Wu J Cong X Wang R Han Y Luo Z Association between allelic variation at the Waxy locus and starch physicochemical properties using single‒segment substitution lines in rice (Oryza sativa L.) Starch-Starke 2013 65 11–12 1069 1077

[B48] Xu F Zhang G Tong C Sun X Corke H Sun M Bao J Association mapping of starch physicochemical properties with starch biosynthesizing genes in waxy rice (Oryza sativa L.) J Agric Food Chem 2013 61 42 10110 10117 24063600 10.1021/jf4029688

[B49] Mikami I Uwatoko N Ikeda Y Yamaguchi J Hirano HY Suzuki Y Sano Y Allelic diversification at the wx locus in landraces of Asian rice Theor Appl Genet 2008 116 7 979 989 18305920 10.1007/s00122-008-0729-z

[B50] Liu A Burke JM Patterns of nucleotide diversity in wild and cultivated sunflower Genetics 2006 173 1 321 330 16322511 10.1534/genetics.105.051110 PMC1461453

